# The Amelioration of the Present Condition of Midwives

**Published:** 1888-01-07

**Authors:** James H. Aveling


### The Amelioration of the Present Condition of Midwives.

By James H. Aveling, M.D., F.S.A.

A paper, bearing the above title, was read before the Hospitals Association by Dr. James H. Aveling. As the subject is one of great interest, and of an importance that can hardly be overrated, we purpose to give, as briefly as possible, the substance of Dr. Aveling's thoughtful and able article. It is the work of a man who has studied the question thoroughly, and has at heart the interests both of midwives and of those entrusted to their care. He has, however, by bitter experience, learned that patience which the unthinking confound with despair ; and while he deplores the lethargy of Parliament, and the slowness of legislative action, he is no less determined than he was thirty years ago to put the status of midwives on a satisfactory footing.

His first statement is the sufficiently alarming one that 4,500 women die annually in childbirth ; two out of every hundred confinements end fatally. As the death-rate among women attended in their confinements by trained midwives belonging to lying-in hospitals—not in the hospitals, but at the patients' homes—is only 1 in 650 ; and in the case of one charity, only 1 in 900, the responsibility of the great mortality already spoken of must lie either with the doctors or the untrained midwives who attend the majority of confinements. Who are likely to deserve it—the doctors who have competent skill and training, or the untrained midwives who have ignorant pretension? Obviously the latter. Besides this annual sacrifice of life, hundreds of women who escape with their lives are, owing to the want of skilful assistance, or to unwarrantable interference during childbirth, rendered useless as workers and wives, and have to pass their existence in continuous discomfort or pain ; in some cases they are left pitiable objects, loathsome to themselves and to all around.

What then, is a midwife ? Briefly, a woman capable of rendering the assistance necessary in normal maternity cases, and of recognising the conditions requiring medical aid. In this definition it will be observed that a midwife is limited in her attendance to normal cases. Upon this point differences of opinion exist. Medical men, as a rule, insist on this limitation, while some ambitious midwifes object to any restriction. Not long since forty-two midwifes signed a paper, which they addressed to the President of the Local Government Board, complaining of the "unsatisfactory professional condition of midwives." Among other matters, it was maintained that a midwife ought to be allowed to treat obstetrical emergencies, and that the limiting of a midwife's practice to natural cases was an evil, inasmuch as the obligatory sending for a medical man lessened her sense of responsibility, while it offended her, and lowered her in the eyes of her patients.

Now the treatment of obstetrical emergencies necessitates the use of instruments and drugs, in fact, it demands the exercise of medical art and science. If midwives wish to attend obstetric emergencies they must qualify themselves to do so, and become medical women. If they cannot obtain a medical diploma, and are not content to limit their calling to attendance on normal cases, the sooner they select some other occupation the better.

The number of midwives at present practising in England and Wales is estimated at 3,000. If we accepted the Continental computation of the number needed we should look upon this as very small. Abroad it is reckoned that there should be one midwife for every 2,000 persons ; according to this calculation there ought to be 15,000 to bear due proportion to our population of 30,000,000. It is probable, however, that all women acting as midwives would not call themselves by that name in filling up the census paper, from which the number of 3,000 was estimated ; and it may be surmised that the probable number of women giving assistance in childbirth in this country amounts to 9,000.

The majority of these have received no special instruction in midwifery. Some of them have taken up their work because their mothers and grandmothers were midwives, and these are the most dangerous, as they have inherited old books on midwifery, and practise according to their teaching. Others having acted as monthly nurses, and having observed what the doctor has done, have undertaken to perform his work when he has not arrived in time. Succeeding in one case, they attempt others, till there comes a difficult one requiring great skill, prompt action, and iron nerve, and with it come catastrophe and death.

There is, however, a large and increasing number of midwives who have studied their work. The existence of this class is due to the lying-in hospitals instituted about the middle of last century, where women can obtain practical instruction in the management of childbirth. More recently the Obstetrical Society of London has done much good by instituting an examination, and granting diplomas to those whom it considers capable of attending natural cases with safety. Unfortunately, the majority of the women who submit to this examination are not of the class most required. The midwives most wanted are those who ply their calling in villages and rural districts, and these cannot afford the fees demanded in the London training hospitals.

At present, in order to provide a sufficient number of competent midwives for this country, there are wanted :—

1. A ready and inexpensive method of instructing women in elementary midwifery.
2. Local examining boards.
3. Licences granted to midwives.
4. Licensed midwives registered.

Every country in Europe except ours has long since made laws and regulations for its midwives, and these laws we have now before us to adopt or avoid as we please. In all countries it is required of a woman before she becomes a midwife that she should be of suitable age, of good moral character, should be able to read and write, and to understand the first four rules of arithmetic. Also that she shall be strong and healthy, and have no physical defects which might interfere with the performance of her duties. In Austria, Belgium, Russia, and Prussia the instruction of midwives is partly paid for by the State. In Sweden and Norway, the Netherlands, and France it is entirely free. In this country it is, as a rule, very expensive, and the whole cost has to be borne by the pupils. In London the charges for instructing midwives are the largest in the world. Queen Charlotte's and the General Lying-in Hospital charge 25 guineas for training and residence; the British Lying-in Hospital charges 22 guineas, and the City of London Lying-in Hospital 20 guineas. In our provincial towns we find an extraordinary difference in the charges. In the Liverpool Lying-in Hospital the pupil midwives pay seven guineas, which includes lodging in the instructing midwife's house during attendance on twenty-five labours. In St. Mary's Hospital, Manchester, one guinea only is paid for attendance on a five months' course of lectures. In the Jessop Hospital for Women, Sheffield, pupil midwives have board and lodging and theoretical and practical instruction in the hospital for twelve months, receiving at the same time a small salary. The result of the high charges in London is the production of a class of midwives not urgently required. No poor woman who would qualify as a rural midwife could afford to pay twenty-five guineas for her training. Why cannot metropolitan lying-in hospitals imitate the good example of similar institutions in the provinces? The method adopted in Sheffield is the most satisfactory. It makes all the difference to a poor woman who has to pay for her instruction, whether she has to give a large sum or receive a small salary during her training. Dr. Keeling, of Sheffield, says: "This method is slow, but the result for our own work has been good. We have no ambition to put a lot of women rapidly through the mill, taking fees and granting certificates, and substituting for the ignorant Sairey Gamp type, an equally objectionable, superficial, and conceited being." There is no reason why pupil midwives should pay such large fees to lying-in hospitals; the necessary instruction can be perfectly well given in the lying-in wards of workhouses.

The second want is *Local Examining Boards.* The Royal College of Surgeons of London having determined that it cannot admit women to the examination for its diploma of licentiate in midwifery, no portal to a legal licence is open to them in England or Wales. In Ireland the power of examining and licensing midwives has long existed, being granted by clause 30 of the charter of the King's and Queen's College of Physicians in 1673. The test examination open to midwives in England is that of the Obstetrical Society of London, which established an examining board fifteen years ago as a tentative measure. The council felt that the work did not properly belong to a scientific society, but thought it a duty to supply an independent examination and reliable diploma to those women who wished to hold some authoritative guarantee of their knowledge of midwifery.

The following scheme for examining midwives has been proposed in the Bill, which we hope some day or other to get passed. England and Wales are to be divided into examination districts, with an examination town in each district. This will save poor women the expense of travelling long distances. The Midwifery Board is to appoint local examining boards, each to consist of five medical men, who shall examine persons desirous of obtaining certificates of fitness to practice as midwives. The fee for examination is not to exceed £2.

Thirdly, *licences must be granted to midwives.* At present it is impossible for a midwife to obtain a licence to follow her calling in England and Wales. Various diplomas and certificates are granted, but no body has the power of conferring a legal licence. The way in which the Act proposes to do this is as follows: It provides that when the board of examiners are satisfied that any person examined by them has been sufficiently instructed in midwifery, and has shown by examination that she is qualified to practise as a midwife, they shall grant her a certificate which shall entitle her to be registered under the Act.

This brings us to our fourth want—the *registration of midwives.* There are now only two registers of midwives; one is to be found in the trades division of the "London Directory," where a list of about sixty midwives, certificated or uncertificated, may be found; the other is kept at the Midwives' Institute and Trained Nurses' Club, but this contains only the names of those who have passed the examination of the Obstetrical Society. These registers are of little use to the general public. The Bill deals with this important question in the following way: A general registrar and local registrars are to be appointed. An alphabetical list of all midwives registered under the Act, to be called the Midwives' Register, is to be printed and published once a year. After the passing of the Act no person shall take or use the name of midwife unless she be registered, and no person shall be eligible for any public appointment who cannot produce her certificate of registration. It is wise to insist only upon the prevention of the use of the title "midwife" by those unregistered, for if we excluded from practice all but registered midwives, we should have to provide a sufficient number of competent women to take the places of those we had deprived of practice. On the Continent, where no one can act as a midwife without being registered, the country is divided into districts, to each of which a midwife is appointed, who is paid a fixed salary. We are not prepared to adopt such a system as this, and must therefore content ourselves with the plan of registration proposed in our Bill.

The first proposal for improving the condition of nurses was made in 1547. About twenty years since the present movement commenced in good earnest, and a most healthy and promising fact relating to it is that it originated with the midwives, and has been persistently supported by them all through. The first impetus to this modern effort was given in 1864 by an association of women calling themselves the "Obstetric College." An "Association of Midwives" also took up the subject, and worked at it energetically. More recently the Midwives' Institute and Trained Nurses' Club has been working energetically in the same direction. In 1870 the Obstetrical Society of London began to consider the the condition of midwives, and instituted its examination. In 1873 the British Medical Association gave its powerful assistance, and a year later the Social Science Association offered its support. From all these bodies deputations have at various times been sent to Downing Street for the purpose of inducing Government to assist in enacting laws for the regulation of midwives. These deputations are courteously received by the Ministers in office, and assured of sympathy and support; then, at the end of the session, comes the announcement that it is impossible to deal with the midwife question at present.

Dr. Aveling then quoted two instances of the mischief done by incompetent midwives, resulting in one case in an infant dying of hæmorrhage, and in the other in the death of both mother and child after terrible suffering. These examples fully proved the importance of the cause he was advocating, and justified his assertion that not only Ministers and Members of Parliament are responsible for the present deplorable state of affairs, but "every English man or woman who has a vote, or can influence a voter; every one who has a tongue, or a pen, and allows it to remain idle." Then in a few words Dr. Aveling appealed to his audience to do all they could in clearing away what he called, with some justice, "this blackest of our disgraces."

